# The Correlation Between Temperament and Fitness for Work According to the Persian Medicine Viewpoints

**DOI:** 10.31661/gmj.v12i.2934

**Published:** 2023-12-17

**Authors:** Mohammad Mahdi Mazhari, Mohammad Reza Shams Ardekani, Mehrdad Karimi, Saber Mohammadi, Mohammad Hossein Ayati

**Affiliations:** ^1^ Department of Traditional Medicine, School of Persian Medicine, Tehran University of Medical Sciences, Tehran, Iran; ^2^ Department of Traditional Pharmacy, School of Traditional Medicine, Tehran University of Medical Sciences, Tehran, Iran; ^3^ Department of Occupational Medicine, Faculty of Medicine, Iran University of Medical Sciences, Tehran, Iran

**Keywords:** Persian Medicine, Occupational Medicine, Temperament

## Abstract

Working is an inseparable part of a human being’s life, every person must have a job to earn a living. However, working comes at a physical and mental cost not only to the working person but also to his/her co-workers and employers, and could also affect the environment. Therefore, each potential employee must be assessed to see if they are suitable for the job and vice versa. In occupational medicine, fitness for work is now determined by clinical and paraclinical testing, which is seen as a useful procedure, yet illnesses and accidents still happen at work. This procedure could be facilitated and enhanced by a more all-encompassing strategy, such as the temperamental theory in Persian medicine. Through temperamental theory, each person is assessed based on specific signs and symptoms according to the qualities of hotness-coldness, and wetness-dryness which can provide extra insights into an employee’s capabilities. This study aims to provide specific characteristics of each temperament, and their relationship with personal traits that could affect job performance, as well as job suggestions for each temperament through an extensive review of Persian medicine books and matching them with the current occupational medicine resources.

## Introduction

Fitness for work is one of the essential topics in the occupational medicine. Its main objective is to conduct a medical examination of the employee and determine whether or not s/he is totally suitable for the job s/he desires and does not constitute a threat to themselves or others [1][2]. In case of job mismatch, society and the organization will suffer huge costs due to reduced efficiency, the possibility of accidents, and the occurrence of physical and mental illness [1]. To achieve the best job, fit and to choose the best person for a job, logical methods and models are used. Currently, the most common method of fitness for work assessment is through occupational medicine models, and standards. In this method, the job requirements, and factors that influence performance are examined to check fitness for work. For job requirements, items like the type of work (physical or intellectual activity), workplace hazards, social and organizational factors (working alone or in a group and interacting with society), ergonomic factors, traveling, and work shifts are considered. Various factors that directly or indirectly affect job performance include a person’s health status, sex, anthropometrics, age, nutritional factors, stress and mental state, sleep disorders, type of work, fatigue, environmental factors such as cold, heat, and pressure (high-pressure environments such as diving and low-pressure environments, such as working at heights), sound, vibration, and environmental pollution are investigated [2].

In short, based on occupational medicine principles, physical health, mental health, physical ability and strength of the person, and environmental factors, including hazards, should be thoroughly examined [1][2][3]. One of the critical points that should be considered during a job suitability assessment is an individual’s personality. As a result, several viewpoints about the relationship between personality and job readiness have been put forward. In the Job Preference Meter, psychologist Frederic Kuder divided personality and job readiness into 10 categories [4]. According to Stephen P. Robbins, it is obvious that individuals have diverse personalities and that different occupations share some traits [5]. Based on this method, many efforts were made to assign people to suitable jobs. Isabelle Myers and her mother, Catherine Briggs, have a unique view on fitness for work, and personality and have tried to suggest a suitable job for each personality using the Myers-Briggs Type Indicator (MBTI) test [6].

Despite the considerable achievements of occupational medicine scientists and the research in this field, considering the broader dimensions of human health and finding ways to explain, and define new and efficient models is required. One of the valuable sources for finding alternative models is using traditional holistic medicine, which classifies people based on their characteristics. Even today, many of these models are used. For instance, the ancient Greek and Roman physicians Hippocrates (460-370 BC) and Galen (129-216 AD), respectively, believed that temperament affected a person’s personality and character [7]. Jung’s two introvert and extrovert types, as well as the theories of Hippocrates and Galen, were combined in Eysenck’s suggested model of personality typology, to which he added neurotic stability and instability [8]. Therefore, it seems that some ancient medical schools, such as Persian and Chinese medicine and Ayurveda, which have their roots in history, could offer valuable insights into fitness for work, which can be used by updating, combining, and matching with the science of occupational medicine, to determine the best job match for each person. These medical schools usually propose a comprehensive, and holistic approach to the human being and the environment, arguing that each person has unique characteristics to differentiate them from others [9][10].

Persian medicine is one of the medical schools with a history of several centuries. Persian medical scholars, including Ibn-e Sina, are among the most outstanding Iranian medical scholars whose Canon of Medicine has been taught in European universities for many years. To prescribe medicine, and provide treatment, in addition to checking the person’s characteristics, Ibn-e Sina paid particular attention to many things, including the job, and always investigated the role of the job in the occurrence of the disease [9][10]. Paying attention to the unique peculiarities of the patients is one of the cornerstones of Persian medicine. The term of temperament is used to illustrate these qualities [11]. Different individual anthropometrics, phenotypic, and personality markers have been used to describe the specific circumstances of each temperament [12].

According to a person’s temperament, Persian medicine offers a variety of treatments for preserving health, including fitness for job [13]. This personality categorization in Persian medicine is based on phenotype, and objective manners of the persons and therefore is rooted in the genotype of the people. Moreover, it is developed during history generation by generation and reaches a rational and practical model. This historical experience could be beneficial for current society by presenting a new approach to help occupational medicine find better and more efficient ways to fit for work assessment.

Therefore, considering the importance of fitness for work and recruitment in occupational medicine, in addition to physical and mental performance examinations, temperamental characteristics can be assessed, and the employees can be recruited based on their temperament [14]. Therefore, the capacity of Persian medicine can be used to improve and facilitate fitness for work assessment and increase work productivity and efficiency. This article aims to discuss the role of Persian medicine, and its proposed insights for fitness for work.

Persian Medicine

The roots of Persian medicine go back to the pre-Islamic era [15]. However, it flourished during the Islamic period. Many scientists, such as Ibn-e Sina (980-1037 AD), the author of Canon of Medicine, Razi (854-927 AD), the author of Al-Hawi, Akhwini (died 983 AD), the author of Hidayat al-Muta’allimin, Jurjani (1040-1136 AD), the author of Zakhira Khwarazmshahi, Ali bin Abbas (930-994 AD), the author of Kamel Sana’a Tabiyeh Al-Malki, and Al-Zahrawi (936-1013 AD), the author of al-Tasrif, significantly contributed to the medical knowledge during the 9th-13th centuries AD, which is known as the golden age of the Islamic civilization. Many books by Iranian scientists of this era, such as Ibn-e Sina’s Canon of Medicine, were taught in Western and Eastern universities until the 17th century. Following the Renaissance, the holistic theory of Persian medicine (temperament theory) gradually lost its position and was replaced by molecular theory and conventional medicine [16]. For the first time, Iranians introduced a temperamental approach to medicine. Later, this word became a universal word in ancient Iran and the Middle Ages [17]. Temperament is divided into nine types; eight non-moderate types which consist of four singular including warm, cold, wet, and dry, and four combined types including warm and wet, warm and dry, cold and wet, cold and dry, and finally, the moderate type [18]. Four combined modes of temperament, including warm and wet (dam/blood), warm and dry (safra/yellow bile), cold and wet (balgham/phlegm), and cold and dry (soda/black bile) and their relations with fitness for work are discussed.

Temperament Theory in Persian Medicine

Medical and therapeutic remedies in traditional Persian medicine are planned, and provided based on each person’s physical and mental characteristics, called temperament. Temperament (mizaj in Persian) is one of the fundamental concepts of Persian medicine that plays a crucial role to provide the preventive and therapeutic health recommendations [19][20]. In this approach, each individual presents specific signs, and symptoms according to the qualities of hotness-coldness and wetness-dryness [21][22]. These signs or symptoms, the most significant of which are touching the skin and detecting apparent coldness or warmth, anthropometrics of the body, the person’s sleeping position, the color, smell, and nature of body secretions, muscle condition and the presence of body fat, reaction to food consumption, color, consistency, and growth of hair, personality traits, speed of movement, and reactions, can all be used to determine the temperament type [22].

## Materials and Methods

Various words were used for ‘work’ in Persian medical books. First, the words job and occupation were searched in several dictionaries, including Dehkhoda, Nafisi, and Burhan-i Qati. Then the words equivalent to ‘work’ were extracted. The most common equivalent words included: profession, occupation, vocation, job, and career. Thus, using Noor® software, an updated and comprehensive software for traditional medicine books, an investigation was done regarding the literacy of different words for ‘work.’ Then, all works (modern and traditional) were reviewed in the software. Several books that have researched old jobs, such as Municipal Ordinances [23], the Encyclopedia of Traditional Jobs [24] and the Guilds from the Constitutional Revolution to the Extinction of the Qajar Dynasty [25] were thoroughly examined as well. The list of jobs and their risks was prepared.

Whenever a specific job or an occupational disease was mentioned, they were further assessed for fitness for work. Moreover, the diseases caused by the lack of fitness were studied in terms of various diseases in traditional medicine, especially dystemperament diseases, as well as their complications, which can affect a person’s health and work. Finally, the results were compared with occupational medicine sources and where they aligned. Ultimately, the following methods and tools were used to collect the data: Specialized books on occupational fitness in occupational medicine, traditional medicine books from the 10th-20th century.

## Results

In Tables-1and2, the temperament types and their relevant characteristics (signs and symptoms) are specified [26][27][28][29][30][31].

Relationship between Temperament and Personality Traits

Each temperament has its personality traits, which are presented in Table-3 [26][27][28][29][30][31].

Fitness for Work based on Different Temperaments

Based on the classification of individuals based on different characteristics in these four categories (temperaments), by determining the temperament of a person, the suitable job for him/her could be predicted. Some examples of these fitness-for-work suggestions for each temperament are mentioned in Table-4.

## Discussion

**Table:** Table1. Temperament Types and Their Visible Characteristics

Temperament type	Visible characteristics (signs)
Warm and wet	Anthropometry: Big body with muscle predominance, big chest, big eyes, big limbs, prominent and obvious joints, square palm. Vascular: Prominent strong pulse, soft pulse, soft veins. Touch: Warmth and softness to the touch of body and limbs, excessive discharge from the eyes. Color: Red and inflamed skin, eyes, and tongue. Hair: Soft and abundant, but hair loss happens.
Warm and dry	Anthropometry: Thin body with few muscles, small eyes, thin limbs, and small and hidden joints. Vascular: Strong pulse, stiff and fast pulse. Touch: Warmth and stiff when touching the body and limbs. Color: Sunny yellow skin, eyes, and tongue. Hair: Very fast hair growth, thick, curly, and black hair.
Cold and wet	Anthropometry: Large body with a predominance of fat and loose flesh, large eyes. Vascular: Soft pulse, soft and slow pulse. Touch: Coldness and softness in the body and limbs when touching the body, loose and hanging skin. Color: White-moonlight skin, white eyes, and tongue. Hair: Thin and smooth hair, premature graying of hair.
Cold and dry	Anthropometry: Thin body, small chest, small limbs, small and hidden joints, small eyes. Vascular: Hidden and narrow veins, weak and stiff pulse. Touch: Coldness in the skin and limbs, dry skin. Color: Dark color and sometimes dark spots on the skin, whiteness and lack of blood vessels in the eye conjunctiva, dark tongue. Hair: Full of hair, but turns white quickly.

**Table:** Table2. Classification of Temperament Types and Their Symptoms

Temperament type	Symptoms
Warm and wet	Sleep: Sometimes dozing and yawning, average sleep. Digestion: Proper digestion, prone to bleeding gums, nose, and anus. Season: Intensification of symptoms in Spring.
Warm and dry	Sleep: lack of sleep, falling asleep late, and very light sleep. Digestion: Constipation, bitterness in the mouth, pungent, and smelly stools, excessive thirst, dry mouth and tongue, dry nose. Season: Aggravation of symptoms in Summer and enjoying the cool and cold air of Winter. Other cases: Strong five senses, low but smelly body secretions such as urine and sweat.
Cold and wet	Sleep: Oversleeping, falling asleep early, and heavy sleep. Digestion: Loose and soft stools, odorless stools and usually white in color, a lot of sticky saliva, low thirst, reflux. Season: Aggravation of symptoms in the Winter and enjoying the Summer. Other cases: Slow five senses, a lot of secretions but without odor, dullness.
Cold and dry	Sleep: Lack of sleep, falling asleep late at the beginning and during sleep, light sleep with nightmares. Digestion: Constipation, false appetite. Season: Exacerbation of symptoms in Autumn and improvement of symptoms in Spring.

**Table:** Table3. Temperament Types and Their Relevant Personality Traits

Temperament type	Personality trait
Warm and wet	Warm and more extroverted: Warm-hearted, strong public relations, willing to work outdoors, interested in the cooperative and group jobs, adventurous, very curious, fun and loving, very sensitive, kind, compassionate and helping, friendly, social and interactive. Behavior: Brave and sometimes bold, risk-taking and competitive, patient, energetic and powerful, tireless, open to criticism, cheerful at work and not bored. Speech: Good expression and logic, strong and loud voice. Thinking: Proper concentration, often thoughtful, clever with a lack of stability in judgment, good memory, and strong retention.
Warm and dry	Extroverted: Warm-hearted, average public relations. Behavior: Speedy in decision-making and taking quick action, hasty with anxiety in behavior, realistic and pragmatic, brave and bold, quick-tempered, accurate and punctual, dry and inflexible, disregarding the judgment of others, high self-confidence, active but getting tired quickly, has an independent, powerful, reliable and hardworking personality. Speech: Fast and continuous. Thinking: Planning with high analytical power, a little difficulty in learning, good memory, very strong concentration, logical, detail-oriented.
Cold and wet	Cold and more extroverted: Empathetic and kind, but less involved in social activities. Behavior: Patient, cool-headed, flexible, quick to change their mindset, quick to be provoked, hard to start an activity, but if started, they will continue it for a long time. Speech: Speaking quietly and slowly. Thinking: Poor memory and slow understanding, lack of planning, expedient.
Cold and dry	Cold and more extroverted: Empathetic and kind but less involved in social activities. Behavior: Patient, cool-headed, flexible, quick to change their mindset, quick to be provoked, hard to start an activity, but if started, they will continue it for a long time. Speech: Speaking quietly, and slowly. Thinking: Poor memory and slow understanding, lack of planning, expedient.

**Table:** Table4. Fitness for Work Suggestions for Each Temperament

Temperament type	Proposed jobs
Warm and wet	Managers, heavy-contact sports such as wrestling, weightlifting, and endurance running, drivers, economic managers interested in discovering theoretical scientific issues, bank clerks, designers, policymakers, doctors and nurses, social workers, teachers, biologists, and nature lovers.
Warm and dry	Fighter pilots, commanders, sprinters and taekwondo athletes, racing drivers.
Cold and wet	Secretaries, working in lonely environments with a lack of communication, archivers, and cohort researchers.
Cold and dry	Poets, politicians, inspectors, inventors, working with precise tools, financial inspectors, accountants, detectives, philosophers, and political analysts.

Work is generally harmful, and associated with many risks. It may cause physical or psychological damage to the working person [32]. If one is working at a job that is physically and mentally suitable, these risks would be minimized and the efficiency will increase. These mental, and physical characteristics of the person are derived from his/her genotype.

It is possible to obtain complete and accurate information about a person through genotype and gene sequence analysis. However, in most cases, it is not practical, and sometimes, scientists have yet to discover all the features of a gene [33]. Each person’s phenotype, which is comprised of their personality and behavioral traits, is derived from their genotype. One can accurately predict the genotype using the phenotype with a high degree of confidence. The potential of identifying an individual’s fixed, unchanging traits with high accuracy and reliability is made possible by the existence of personality and behavioral traits [34]. Currently, there are several methods to check fitness for work, the most common and scientific of which is using clinical and para-clinical measures based on the principles of occupational medicine [35]. In this method, the person goes via a physical examination and in some cases, their personality is examined through a structured psychological questionnaire [36]. However, humoral (temperament) theory in Persian medicine can be considered an alternative method. By examining temperament, Persian medicine evaluates and predicts the physical characteristics, personality traits, behavior, and abilities of an employee. In addition to the above, according to the type of temperament and environmental conditions, Persian medicine may suggest measures for better work performance and disease prevention [37]. Nevertheless, should the job match the principles of Persian medicine and the temperamental approach, work performance will improve, physical and mental damage will be minimized, and the work will be enjoyable. Also, compliance with these principles for employers will minimize disease burden, especially occupational diseases, and increase productivity [2]. Another advantage of this approach would be talent search, which can identify children and adolescents’ talents and guide them to the right job [38]. In general, it is possible to suggest ‘the Persian medicine fitness for work’ model as a complementary method to the assessment principles in occupational medicine. Based on Persian medicine, individuals with a warmer temperament usually have excellent public relations in terms of their extraversion characteristics. Because of this, they are competent group leaders and excellent managers. However, they are unsuitable for professions demanding close attention to detail, working alone, or in a permanent setting. Warm-dry temperament types react quickly and sharply, but they also function within a set framework and structure. Because of their boldness and quickness in action, they are good candidates for leadership positions. Those with a colder temperament have slow movements but are patient and suitable for jobs, such as archiving and follow-up of cohort studies. People with a cold-dry temperament are very meticulous and precise. These people have a good sense of responsibility and are suitable for jobs that require intellectual activity. So, they are suitable for jobs such as an inspector, detective, and accountant. It should be noted that work affects temperament, and it is crucial to take measures to create a work-life balance and prevent the occurrence of subsequent diseases related to temperament. For example, a person with a colder temperament is suitable for the job of answering the phone in a quiet environment. However, by implementing measures such as warming the environment, increasing family communication, ingesting warm foods, and recommending appropriate exercises, diseases associated with cold and dryness could be prevented. On the other hand, a person with a warm and dry temperament is suitable for jobs with high activity and speed, yet this job will cause extra heat and dryness. So, s/he is recommended to prevent further problems by drinking more fluids, working in cold environments, and avoiding warm and dry foods.

This theory needs further investigation to evaluate its efficacy in practice. To further examine the relationship between temperament and fitness for work, the authors would suggest the following topics for future studies:

1. The relationship between temperament and job success in various jobs,

2. The relationship between temperament and examination of physical and mental complications in jobs,

3. Prevention of occupational diseases using Persian medicine according to the individuals’ temperament and the job temperament.

Currently, fitness for work in occupational medicine is first examined based on the type of occupation of the person, and then para-clinical examination and necessary tests are performed according to the occupational requirements and hazards of work environment. Each temperament seems to have unique athletic prowess, anthropometric qualities, and unique psychological traits. It is possible to leverage a person’s temperamental traits and qualities to make certain tasks more effective for them or to make other jobs for them less beneficial or even dangerous owing to situations like fatigue. To increase efficiency and productivity and prevent accidents, it is suggested to use Persian medicine’s temperamental recommendations as a complementary method to employment examinations and common fitness for work.

## Conclusion

Persian medicine is one of the old schools of holistic medicine. In Persian medicine, each person is assessed through their unique physical and mental characteristics, known as temperament, which can be determined via certain signs and symptoms. The Persian physician may recommend a better employment fit using the temperamental method. Along with the concepts of occupational medicine, information on one’s temperament may serve as the foundation for establishing fitness for work. By combining the fitness for work model in Persian medicine and the principles of occupational medicine, people with higher capabilities are employed who are less prone to occupational disease and dissatisfaction and show more flexibility. They will be more productive and efficient which could help promote the organization.

## Conflict of Interest

The authors declare no conflict of interests.
